# Translating Big Data into Smart Data for Veterinary Epidemiology

**DOI:** 10.3389/fvets.2017.00110

**Published:** 2017-07-17

**Authors:** Kimberly VanderWaal, Robert B. Morrison, Claudia Neuhauser, Carles Vilalta, Andres M. Perez

**Affiliations:** ^1^Department of Veterinary Population Medicine, College of Veterinary Medicine, University of Minnesota, St. Paul, MN, United States; ^2^Informatics Institute, University of Minnesota, Minneapolis, MN, United States

**Keywords:** animal movement, big data, machine learning, modeling and simulation, surveillance

## Abstract

The increasing availability and complexity of data has led to new opportunities and challenges in veterinary epidemiology around how to translate abundant, diverse, and rapidly growing “big” data into meaningful insights for animal health. Big data analytics are used to understand health risks and minimize the impact of adverse animal health issues through identifying high-risk populations, combining data or processes acting at multiple scales through epidemiological modeling approaches, and harnessing high velocity data to monitor animal health trends and detect emerging health threats. The advent of big data requires the incorporation of new skills into veterinary epidemiology training, including, for example, machine learning and coding, to prepare a new generation of scientists and practitioners to engage with big data. Establishing pipelines to analyze big data in near real-time is the next step for progressing from simply having “big data” to create “smart data,” with the objective of improving understanding of health risks, effectiveness of management and policy decisions, and ultimately preventing or at least minimizing the impact of adverse animal health issues.

## Introduction

As our capacity to collect and store data continues to expand rapidly, challenges in veterinary epidemiology are shifting from data acquisition to translating data into meaningful insights about animal health. While human medicine and public health have harnessed big data to optimize “precision” care and track trends in human diseases (1–8), big data in the field of veterinary medicine have been mostly focused on spatial analyses and bioinformatics (9–13). However, the use of big data for animal disease surveillance is a rapidly growing field (14, 15). The promise of big data, as has been witnessed in areas ranging from human health to business and marketing, is the capability to target specific populations and track or even anticipate trends (16). The development and refinement of such capabilities in veterinary epidemiology could significantly improve our ability to identify and respond to emerging animal health concerns, especially if collection and analysis of data occurs in near real-time rather than retrospectively.

**Figure 1:**
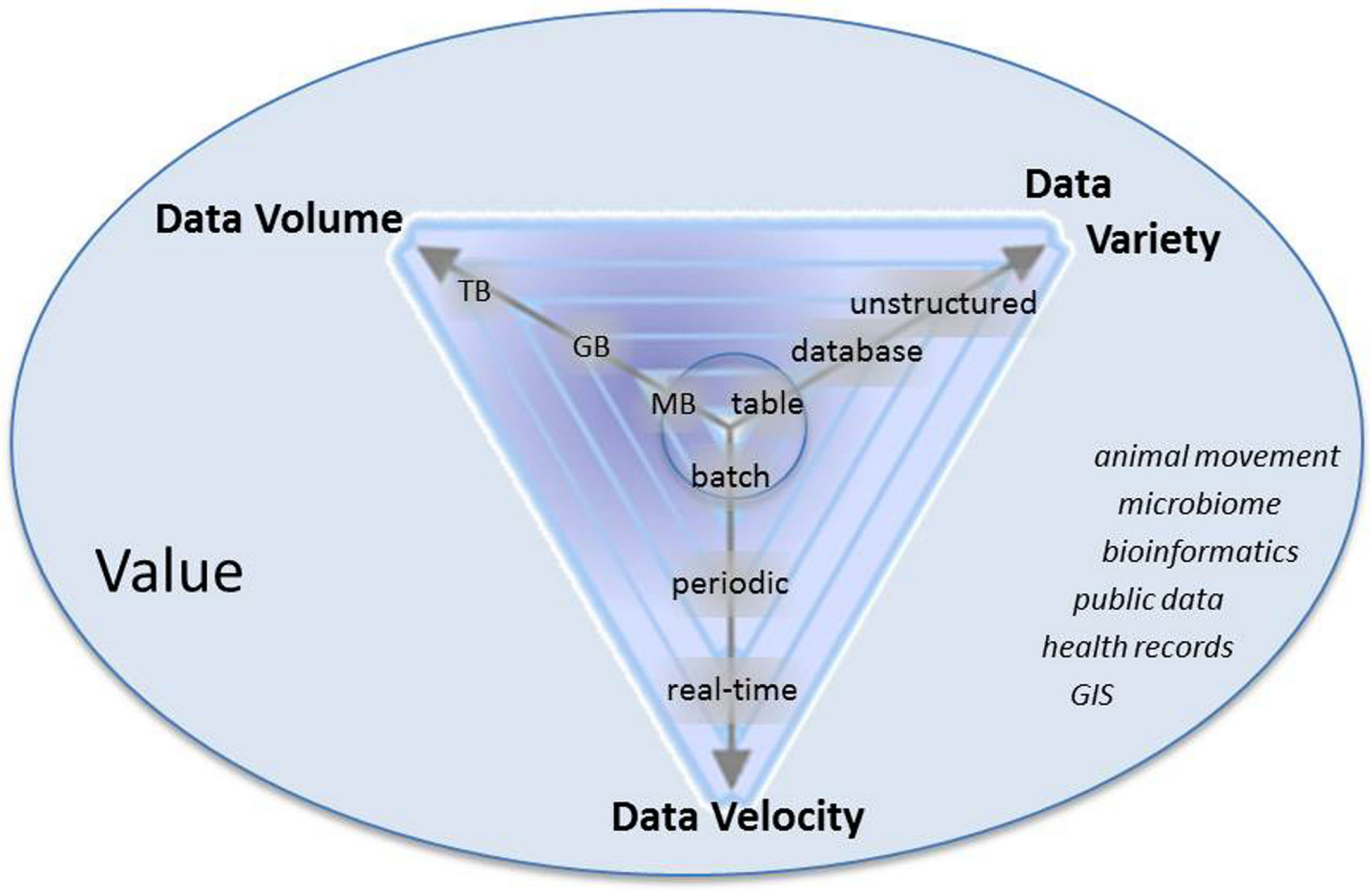
Characteristics of big data: volume, variety, velocity, and value. Arrows represent that data are progressively getting larger (more volume), more variable, and are accruing at faster rates than historically in the field of veterinary epidemiology. Italicized words are examples of types of data in veterinary epidemiology that meet some combination of volume, variety, and velocity.

Big data typically have certain characteristics, referred to as the four “V’s” (Figure 1) (16, 17): *Volume* refers to the size of the dataset, which is typically an order of magnitude or more than what has previously been available within a given field (10); *variety* refers to different forms of data that may have been generated for different purposes or collected at different spatial or temporal scales; *veracity* addresses uncertainties in data; and *velocity* refers to the rate at which data are accrued. High velocity data should not be thought of as a “dataset,” but rather a “data stream” (17, 18). Applying analytics to volume, variety, veracity, and velocity generates a fifth “V”: the *value* of big data to create novel insights and inform decision-making. The analysis of big data, as applied to veterinary epidemiology, is not fundamentally novel compared to traditional or historical practices, but rather differs in complexity, scale, and scope.

Veterinary epidemiological data that are or are becoming “big” include “-omics” data, geospatial data, publically available data repositories such as World Animal Health Information System^1^ and EMPRES Global Animal Disease Information System (Empres-i^2^), clinical data or digitized health records from both companion and food animals, data on animal movement from local to international scales, and production data from food animal industries (Figure 1) (14, 15, 19). The analysis of such data can be used to understand health risks and minimize the impact of adverse animal health issues by, for example, increasing the effectiveness of control and surveillance by identifying high-risk populations through the analysis of spatial and animal movement data; combining disparate data or processes acting at multiple scales through epidemiological modeling approaches; and harnessing high velocity data to monitor animal health trends and for early detection of emerging health threats.

Generating and storing big data are becoming increasingly easy, but we now face challenges in translating the abundance of available data into meaningful information. This challenge, combined with the capability to analyze epidemiological patterns in near real-time, creates a need to develop effective tools and data pipelines to move from simply having “Big Data” into the creation of “Smart data.” Using the four V’s as an organizing framework, we review recent examples of big data analytics and highlight insights gained from approaching veterinary epidemiology with a big data perspective.

## Volume: Identifying High-Risk Populations with Big Data

Collecting and analyzing very large data sets has become increasingly common as technologies for storage and computation advance. For example, research utilizing bioinformatics approaches, detailed data on the demographics and movements of animal populations, and large scale spatial datasets routinely generate terabytes of data, stimulating a new frontier of advanced analytics to handle such data (9–11). Here, we do not provide an exhaustive review of the use of high volume datasets in veterinary epidemiology, but rather select a few diverse examples that highlight the potential use of big data to identify high-risk populations.

Risk of infection is rarely homogenous in a population, and the ability to identify heterogeneities in risk allows for targeted surveillance and control measures. Risk-based strategies are typically more cost-effective than non-targeted strategies, both in terms of early detection and rapid control of a disease (10, 20–22). Because movement of animals between locations is a key risk factor for many infectious diseases, many countries now implement mandatory animal traceability programs (23–26). For example, national or multinational programs, such as the European Union’s Trade Control and Expert System and the United Kingdom’s Cattle Tracing System, track shipments of production animals across space and time, generating a rich source of information for rapid response to health threats (27–29). In the absence of national regulatory frameworks, large production companies often keep records on the movement of animals between company farms (30). Movement data from a single swine production company in the US contained information on the origin and destination of 9.1 million pigs annually, totaling ~25,000 per day. Such databases can be used to construct contact networks that represent potential transmission pathways in a population, and social network analysis can be used to quantify the connectivity of each node within the network and to assess the population’s vulnerability to infectious disease epidemics (26, 31–33). Identifying premises that likely play critical roles in disease spread, such as highly connected farms or farms lying on bottlenecks within the network, can inform control measures that are more effective at limiting disease spread than non-targeted approaches (10, 21, 25). Given the high velocity nature of animal movement data, it is relatively easy to envision how risk estimates could be updated in near real-time, provided that data are efficiently captured in the field, analyzed, and reported to decision-makers.

Substantial spatial heterogeneities exist in the occurrence of infectious diseases, and management and analysis of large spatial datasets represents another facet of voluminous data (34). Numerous spatially explicitly datasets exist for environmental and climatic factors [e.g., Ref. (35)], land cover and use [e.g., Ref. (36, 37)], distributions of at-risk, reservoir, and vector populations [e.g., Ref. (37–39)], and satellite imagery and remote sensing products (40). In addition, the increasing use of GPS tracking devices creates a rich source of data on the movement of people, vehicles, and animals that can be used to dynamically represent exposure and transmission dynamics (41). These data can be combined with geo-referenced disease data [e.g., Ref. (42)] to identify environmental correlates of disease through ecological niche modeling, thus contributing to our ability to understand and map a pathogen’s geographic distribution (9, 43–45). By utilizing near real-time updates in environmental data and locations of new cases, risk maps can become evolving rather than static representations of risk (43, 46). Remote sensing, in particular, could be re-framed as a high velocity source of data, as many satellite-based data are updated at regular intervals (43). Ultimately, the ability to predict the occurrence of pathogens through space and time will allow for more effective targeted surveillance and control.

## Variety: Combining Disparate Data

The challenge emerging from the need to assemble datasets from multiple, disparate sources is not new within epidemiology. Analysis of such data is complicated in that data are often aggregated at different spatial and temporal scales, and datasets must be aggregated or disaggregated to harmonize the spatiotemporal scale of the consolidated dataset. Even when combining a single type of data (i.e., diagnostic records) from various institutions, inconsistencies in data structure and vocabulary must be mapped to make the data interoperable.

The use of universally recognized data formats is important for enhancing connectivity of data between different sources (e.g., different laboratories and clinics) (47, 48). Human diagnostic laboratories have long had standardized vocabularies for health records, such as HL7, LOINC, and SNOMED, but standardized vocabularies are typically underused in veterinary medicine (49). To overcome this challenge, the Clinical Wildlife Health Initiative has worked to create a standardized terminology for clinical signs in admitted wildlife. This standardization enhances the ability to pool data from multiple clinics into a common dataset, thus creating a powerful network of clinics in which health trends could be tracked (50). Similar efforts are underway for swine diagnostic data (48). Related to the current lack of data standardization, many sources of animal health data are not readably usable in statistical models, such as “unstructured” text-heavy data (15, 49, 51). While it is possible to use text-mining techniques to extract information from unstructured text fields in clinical or diagnostic records (52, 53), homogenizing and naturalizing data into uniform and standardized formats is critical for maximizing accuracy and ensuring smooth automation (8).

Finally, data relevant to disease dynamics are representative of processes that operate at different spatial and temporal scales. Epidemiological modeling provides a means to connect processes across multiple scales and account for the inherent dynamic elements of disease systems. For stochastic disease models, thousands or even hundreds of thousands of simulations are performed to understand the behavior of the system and optimize parameter values, thus requiring extensive computational resources and generating big data. Machine learning techniques, such as random forests and genetic algorithms, are used to optimize parameter values so that the model simulates epidemiological dynamics that closely resemble real-world data. Computational modeling provides an effective means to link data to processes, and understand mechanistically how disparate data may interact to influence the occurrence of disease.

## Velocity: Harnessing High Velocity Data

Of the Vs of big data, velocity represents the largest departure from traditional data processing in veterinary epidemiology, but it also has the most potential for revolutionizing the field, particularly in regard to monitoring and surveillance (2, 3, 7, 15, 19, 54). Sources of high velocity data include digitized records from clinics and diagnostic laboratories, analysis of Google search trends and social media, and mortality and abattoir data (2, 3, 15, 49, 54–56). Analysis of data through time allows for the establishment of baselines to which emerging data can be compared (49). The typical values for a metric that relates directly or indirectly to disease (e.g., incidence or production levels) are summarized for a particular population or spatial location, and deviations outside the normal variation of the metric can be used as an indicator of an outbreak (57, 58). Time-series analysis provides an additional method for mathematically quantifying temporal patterns, incorporating seasonality and long-term trends (58). Short-term predictions can then be made in terms of expected incidence or prevalence over time. Departures from expectations can be considered “anomalies” and may serve as early warnings for emerging threats or altered disease dynamics that warrant further investigation or intervention (54, 56, 58).

For example, a recent initiative involving 700 veterinary hospitals in the US tracked the daily proportion of patients with certain clinical signs, contrasting new data with averages from a retrospective period of time. As a proof-of-concept, the system was able to rapidly detect a simulated outbreak scenario and generate an outbreak alert (19). Similarly, daily condemnation rates of pig carcasses at abattoirs in Canada were evaluated to detect aberrations in the data stream that may signal a disease outbreak. Aberrations detected in a retrospective analysis coincided with several documented disease outbreaks in swine, thus demonstrating the potential timeliness of a syndromic surveillance system based on abattoir data streams (59). Finally, Guernier et al. (54) found that Google search trends could be used to track the occurrence of tick paralysis in companion animals in Australia, and certain search terms could potentially be used as early indicators of high-risk periods. These examples highlight the potential for high velocity and high volume data to enhance surveillance capabilities. Such applications may be particularly relevant for syndromic surveillance, where the causative agent may not be identified and the focus of the analysis is on tracking suites of clinical signs, or a syndrome, that may be associated with an endemic or emerging disease (49).

**Figure 2:**
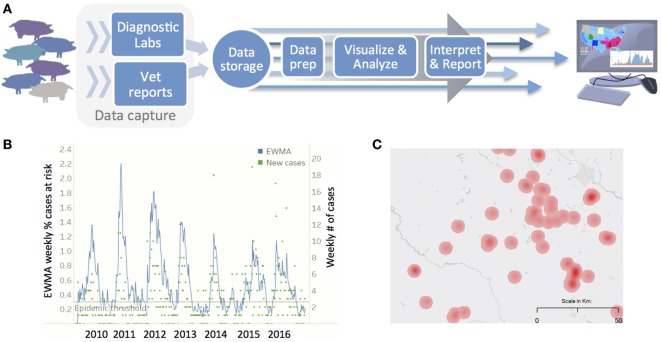
**(A)** Data pipeline utilized by the Morrison Swine Health Monitoring Project for generating near real-time insights about the spatiotemporal incidence of porcine reproductive and respiratory syndrome (PRRS) virus, including weekly reports on the **(B)** incidence of PRRS, with trends reported as an exponential weighted moving average (EWMA), and **(C)** heatmaps of PRRS risk based on the geographic distribution of sow farms shedding PRRS.

The capability to acquire and analyze high velocity data streams requires the development of data pipelines, which can automate and streamline the processing of big data for real-time insights and rapid response. For example, the Morrison Swine Health Monitoring Project (MSHMP) is a high velocity data stream that is effective at tracking the spatiotemporal dynamics of several, high-impact infectious diseases in the US swine industry (60, 61). As of November 2016, MSHMP included data from more than 1,000 sow farms managed by 29 production companies; the status of ~46% of breeding sows in the US are tracked through MSHMP on a weekly base. In this case, the data pipeline involves (a) capture of data from disparate data sources, including veterinarians and diagnostic laboratories, (b) storage and (c) processing data to prepare datasets for (d) data visualization and analysis to (e) ultimately interpret and report up-to-date trends in incidence, prevalence, genetic diversity, and spatial occurrence of swine diseases in the US (Figures 2A–C). This data pipeline is being used, for example, to establish dynamic baselines for porcine reproductive and respiratory syndrome (PRRS) virus incidence, detect the onset of seasonal PRRS epidemics, and provide value to participating producers.

## Creating Value: Big Data Challenges and Opportunities in Veterinary Epidemiology

In the coming decades, the greatest challenge in big data epidemiology will be to move toward creating value. Putting big data to work requires expanding our definition of the V’s of big data to include three A’s: accuracy, accessibility, and automation. While some may purport that the sheer quantity of big data means that inaccuracies in the data are washed out, this may not be true if issues with confounding, measurement error, and selection bias scale with sample size (5, 47, 62). For example, data mining based on Google searches or Twitter may misrepresent the population at risk given biases in internet use. In addition, the value of extracting data from clinical records or diagnostic laboratory databases is dependent on the quality of record keeping and data entry. Thus, error checking and quality control should be incorporated into big data processing to ensure reliability (47). Accessibility it also a critical challenge, encapsulating fundamental concerns related to data confidentiality and ownership (8, 14, 18, 47), data engineering issues revolving around data structure and connectivity (8), and limited availability of trained personnel capable of extracting data from databases (14). Finally, high velocity data create a need to automate data pipelines for routine and repeated use. Automation is key for harnessing big data for monitoring and surveillance (8, 49, 56).

A major criticism of big data analytics is that it lacks the rigor of hypothesis-driven, controlled experiments for determining causation (16, 62). However, correlations identified through the analysis of big data are useful for hypothesis generation and prediction (5, 17). In addition, the increasing numbers of measurable explanatory factors available from diverse sources necessitate the use of relatively new (to veterinary epidemiologists) statistical approaches, such as machine learning, that are more appropriate for handing datasets with a large number of covariates (17, 18, 63–65). Due to the large number of potential variables, care must be taken to identify spurious correlations (5, 66), and the use of large datasets does not necessarily increase a study’s validity. Sound epidemiological principles for the interpretation of observational data are required (62).

As in all long-term monitoring programs, sustainability of big data surveillance and monitoring efforts is a constant challenge (49). For example, voluntary reporting programs such as MSHMP rely on weekly reporting by veterinarians, and adoption of new data standards and sharing of data across organizations requires investment of time, resources, and complicated data-sharing agreements. Even ensuring that all data fields are complete in clinical or diagnostic records (such as location data) requires investment of time and diligence by workers (14, 47). Despite substantial individual and institutional investments, the collective and long-term benefits for big data animal health monitoring at the population, regional, or national level may be murky for the individual practitioner. Thus, sustainability may depend on creating short-term value for participating entities. For companion animal and equine medicine, aggregated health data could be used to research and subsequently deliver “precision” veterinary care that is tailored to the individual (5, 8). For livestock industries, short-term value may focus on research that intends to improve herd and flock management.

The advent of big data has implications for the education of veterinary epidemiologists (6, 13, 17, 51), including technical skills, such as computer programming, that may not be a traditional part of epidemiological training. While epidemiologists may never be responsible for creating complete software applications, the ability to manage relational databases or write simple scripts in a programming language to facilitate preparing data for analysis is critical when datasets become too large to process manually. Further, the analysis of big data often entails the use of supercomputing resources, which usually requires some familiarity with parallel processing and IT systems. To train the current workforce, workshops with hands-on computational activities are needed. Current curricula in graduate education should be expanded to include machine learning as well as traditional statistics, and coding as well as core epidemiological skills. Alternatively, graduate programs in the veterinary sciences could actively recruit students with computer science backgrounds that will readily be able to apply big data thinking to veterinary data. Veterinary epidemiologists with skillsets that allow them to directly engage with, manipulate, and analyze large datasets will be ideally situated to propel veterinary epidemiological research and practice into the coming decades.

The role of big data in veterinary epidemiology, and veterinary medicine more generally, has in some ways been inevitable from the beginning of the digital age, where data have become ever easier and cheaper to generate and store. At this point in time, we are at a turning point in terms of our ability to translate big data, which has existed for well over a decade, into smart data that create meaningful insights for animal health. Forward thinking is required to position our IT systems and workforce to harness the potential of big data. Indeed, from our perspective, big data should not be described as something that exists, but rather a capability. The real promise of big data is to create value out of disparate, chaotic pieces and extract real-time insights from data streams, thus creating a potentially revolutionary opportunity for veterinary epidemiology.

## Author Contributions

KV wrote the perspective. RM, CN, CV, and AP contributed ideas and reviewed the manuscript. CV contributed figures related with the Swine Health Monitoring Project.

## Conflict of Interest Statement

The authors declare that the research was conducted in the absence of any commercial or financial relationships that could be construed as a potential conflict of interest.

## Footnotes

^1^http://www.oie.int.

^2^http://empres-i.fao.org.
